# Setting temporal baselines for biodiversity: the limits of available monitoring data for capturing the full impact of anthropogenic pressures

**DOI:** 10.1038/srep41591

**Published:** 2017-01-30

**Authors:** Jean-Baptiste Mihoub, Klaus Henle, Nicolas Titeux, Lluís Brotons, Neil A. Brummitt, Dirk S. Schmeller

**Affiliations:** 1 UFZ-Helmholtz Centre for Environmental Research, Department of Conservation Biology, Permoserstr. 15, 04318 Leipzig, Germany; 2 Sorbonne Universités, UPMC Université Paris 06, Muséum National d’Histoire Naturelle, CNRS, CESCO, UMR 7204, 61 rue Buffon, 75005, Paris, France; 3 European Bird Census Council (EBCC) and Forest Sciences Centre of Catalonia (CEMFOR-CTFC), InForest Joint Research Unit (CSIC-CTFC-CREAF), Ctra. Sant Llorenç de Morunys km 2, 25280 Solsona, Spain; 4 Centre de Recerca Ecològica i Aplicacions Forestals (CREAF), 08193 Cerdanyola del Vallés, Spain; 5 Consejo Superior de Investigaciones Científicas (CSIC), 08193 Cerdanyola del Vallés, Spain; 6 Department of Life Sciences, The Natural History Museum, Cromwell Road, South Kensington, London SW7 5BD, UK; 7 CNRS, EcoLab, 31062 Toulouse, France; 8 Université de Toulouse, UPS, INPT, EcoLab (Laboratoire Ecologie Fonctionnelle et Environnement), 118 route de Narbonne, 31062 Toulouse, France

## Abstract

Temporal baselines are needed for biodiversity, in order for the change in biodiversity to be measured over time, the targets for biodiversity conservation to be defined and conservation progress to be evaluated. Limited biodiversity information is widely recognized as a major barrier for identifying temporal baselines, although a comprehensive quantitative assessment of this is lacking. Here, we report on the temporal baselines that could be drawn from biodiversity monitoring schemes in Europe and compare those with the rise of important anthropogenic pressures. Most biodiversity monitoring schemes were initiated late in the 20^th^ century, well after anthropogenic pressures had already reached half of their current magnitude. Setting temporal baselines from biodiversity monitoring data would therefore underestimate the full range of impacts of major anthropogenic pressures. In addition, biases among taxa and organization levels provide a truncated picture of biodiversity over time. These limitations need to be explicitly acknowledged when designing management strategies and policies as they seriously constrain our ability to identify relevant conservation targets aimed at restoring or reversing biodiversity losses. We discuss the need for additional research efforts beyond standard biodiversity monitoring to reconstruct the impacts of major anthropogenic pressures and to identify meaningful temporal baselines for biodiversity.

A comprehensive understanding of biodiversity responses to anthropogenic pressures is necessary if human development is to remain within planetary boundaries^1^, and for assessing its impact on biological evolution in the Anthropocene^2^. Temporal baselines are essential for reliably measuring changes in biodiversity over time^3^, for instance by mitigating the consequences of the shifting reference syndrome^4^,^5^,^6^. Further, temporal baselines also frame conservation objectives by identifying the biodiversity reference states aimed for guiding the feasibility of and efforts required to reach those objectives^7^, and by defining the time-period within which progress and change are to be evaluated^8^.

In this respect, the lack of knowledge about biodiversity states prior to the rise of harmful anthropogenic activities is a critical limitation for understanding the full impact of such pressures and, therefore, for implementing appropriate conservation goals and strategies. Failing to set relevant temporal baselines for biodiversity represents a major risk for implementing effective biodiversity conservation. It may decrease our understanding of past and therefore current changes, misinform conservation objectives and restrict our ability to assess progress. Nonetheless, there are several obstacles that limit our ability to define relevant temporal baselines for biodiversity.

Monitoring schemes provide an important source of information on biodiversity change, guiding further research, conservation assessment and planning^9^. Monitoring schemes are typically used to document changes in biodiversity over time, making the implicit assumption that the state of biodiversity when the scheme started is an appropriate temporal baseline against which to measure that change. However, most structured biodiversity monitoring schemes have been initiated within the last few decades, whereas most of the anthropogenic pressures that are currently impacting biodiversity have been operating over centuries or even millennia^10^,^11^,^12^. Current drivers of biodiversity decline, such as habitat loss and fragmentation, exploitation, pollution, climate change or species introductions result from processes initiated long ago by accelerating agricultural, technological and industrial developments, driven by an increasing human population and its societal needs^13^,^14^,^15^,^16^. This mismatch between the restricted temporal coverage of biodiversity monitoring and the long history of anthropogenic pressures inevitably limits any assessment of the full impacts of such pressures on biodiversity^12^,^17^.

Furthermore, the biodiversity data from these schemes remain scattered, suffer from geographic and taxonomic bias and from strong methodological heterogeneity across space and time^18^,^19^,^20^. These issues make such data difficult to access, to assemble and to analyze over large spatial and temporal scales^9^,^21^,^22^. Although significant efforts are underway to mobilize and standardize biodiversity data globally^23^, progress towards the fully operational integration of information across scales is still insufficient to provide unbiased knowledge of the status and trends of biodiversity^24^. The recently proposed Essential Biodiversity Variables (EBVs), encompassing six EBV classes (Genetic composition, Species populations, Species traits, Community composition, Ecosystem function, and Ecosystem structure), provide a framework for comprehensively representing the different components of biodiversity in order to measure change over time^24^,^25^, to identify the most important gaps in data coverage and to improve monitoring practices across time and space^3^,^26^.

Although the limitations of biodiversity information available from monitoring schemes are widely recognized, a comprehensive and quantitative evaluation of the potential of monitoring schemes to identify temporal baselines capturing the impacts of major anthropogenic pressures on biodiversity is still lacking. Yet such an assessment is urgently required as it would help provide stakeholders with precise information on the knowledge gaps in currently available biodiversity data. Here, we conduct such a quantitative evaluation of the temporal baselines that could be identified using comprehensive information on biodiversity monitoring schemes sourced from several meta-databases. We focus on Europe as one of the regions of the world with the oldest and most intensive biodiversity monitoring efforts. We report the start of European biodiversity monitoring schemes to examine the possibilities offered by available data for documenting past states of biodiversity with respect to different (i) taxonomic groups, (ii) EBV classes and (iii) types of data collected. Then, we compare the onset of biodiversity monitoring schemes with historical time-series or reconstructions of the main anthropogenic pressures that are currently acting on biodiversity at global or regional scales. We show that the past biodiversity states that may be estimated from available biodiversity monitoring data are unlikely to reflect the full impact of anthropogenic pressures on biodiversity. We highlight the implications for setting appropriate temporal baselines and the consequences for biodiversity conservation management practices and policies, and we provide recommendations on possible ways to move forward with this.

## Results

### Biodiversity monitoring and the history of major anthropogenic pressures

**Figure 1:**
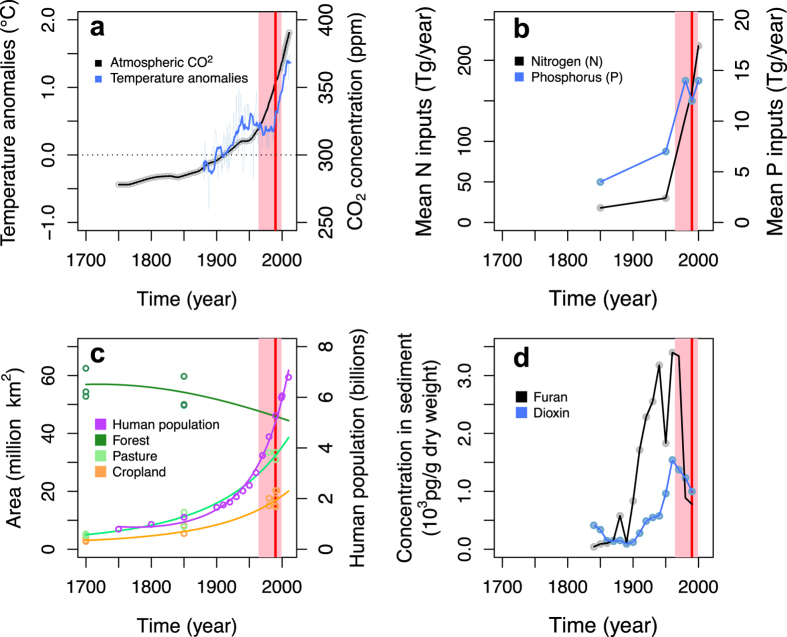
Temporal mismatch between biodiversity monitoring schemes in Europe and major global or regional anthropogenic pressures known to impact biodiversity. The onset of biodiversity monitoring is represented using the median value (vertical red line) and the first and third quartiles (light red area) of the starting years of biodiversity monitoring schemes (see Table 1). Major pressures include (**a**) climate: global temperature anomalies and European atmospheric concentrations of carbon dioxide, (**b**) global anthropogenic nitrogen and phosphorus, (**c**) global human population sizes and global land use changes and (**d**) pollutant emissions in the United Kingdom (UK) (sourced from^52^,^53^,^54^,^55^,^56^,^57^,^58^).

Most of the major anthropogenic pressures that are known to impact biodiversity began hundreds of years earlier than the start of biodiversity monitoring schemes (Fig. 1). In Europe, most of these schemes started in the late 20^th^ century (Fig. 1 and Table 1). Only a small proportion of these schemes were initiated before the middle of the 20^th^ century (c.a. 12.5% before 1950, N = 210) and c.a. 50.6% (N = 857) started 1990 or later. More importantly, anthropogenic pressures started to escalate exponentially from the beginning or the middle of the 20^th^ century, while the vast majority of biodiversity monitoring schemes started only after these pressures had already reached more than half of their present-day order of magnitude or had already peaked and decreased (Fig. 1 and Supplementary Table S1). As a consequence, a large part of the anthropogenic pressures on biodiversity have operated long before any data on the past states of biodiversity was recorded by monitoring schemes in Europe.

### Taxonomic groups

**Figure 2:**
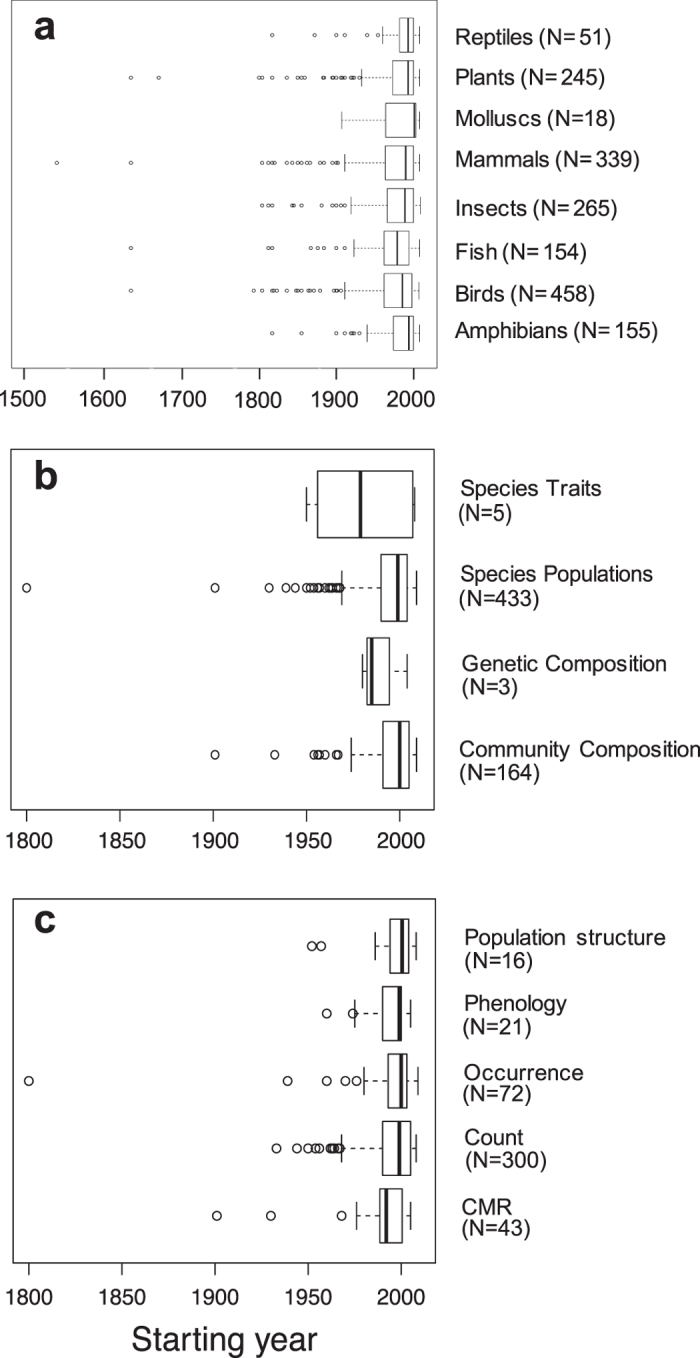
Univariate boxplots based on the starting year of biodiversity monitoring schemes in Europe for each of (**a**) the taxonomic groups studied (from entire database), (**b**) the EBV classes targeted and (**c**) the type of data collected (from reduced dataset using DaEuMon only; see Methods).

**Figure 3:**
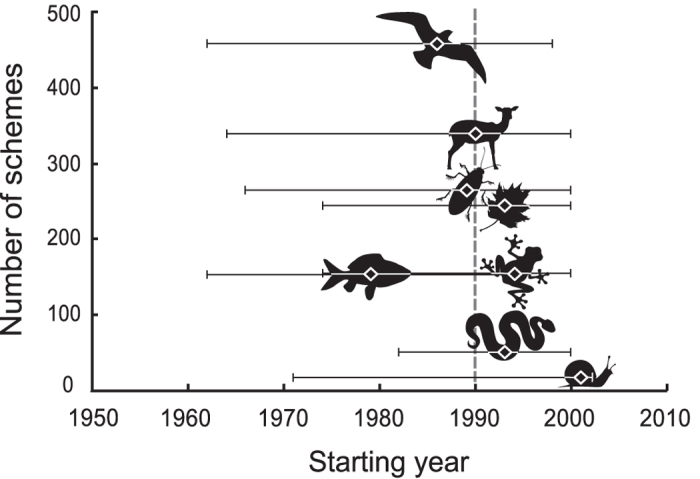
Taxonomic heterogeneity in the start of biodiversity monitoring schemes in Europe (median starting dates ± first and third quartiles) with respect to the number of schemes. The eight taxonomic groups (amphibians, birds, fishes, insects, mammals, molluscs, plants and reptiles) are represented with schematic icons. Dashed line indicates the overall median starting date across all taxonomic groups.

**Figure 4:**
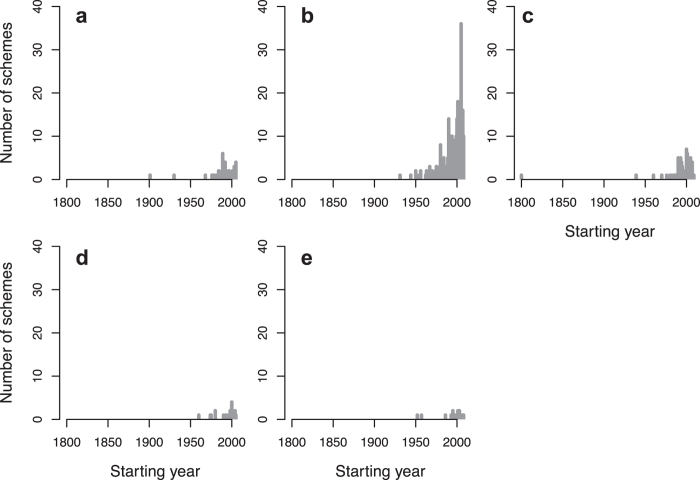
Number of monitoring schemes initiated over time according to their starting year for each taxonomic group studied: (**a**) amphibians (N = 155), (**b**) birds (N = 458), (**c**) fishes (N = 154), (**d**) insects (N = 265), (**e**) mammals (N = 339), (**f**) molluscs (N = 18), (**g**) plants (N = 245) and (**h**) reptiles (N = 51).

Biodiversity monitoring schemes in Europe focus on amphibians, birds, fishes, insects, mammals, molluscs, plants and reptiles (Table 1 and Supplementary Fig. S1). We found strong heterogeneity among taxonomic groups in the start of biodiversity monitoring schemes (Chi^2^_7_ = 33.314, N = 1635, p < 0.001, Figs 2a and 3), with an exponential overall increase in the number of schemes starting from the 1950’s (Fig. 4). In terms of median starting dates, birds and fishes are the focus of the oldest schemes, whereas schemes focusing on amphibians, molluscs, plants and reptiles are more recent (approx. a decade later; Table 1 and Supplementary Table S2). Birds and mammals have been the most common focus of the schemes (27%, N = 458 and 20%, N = 339, respectively). Other taxonomic groups such as amphibians, fish, plants and insects were less studied but reptiles and molluscs were the least monitored groups (3%, N = 51 and 1%, N = 18 respectively; Table 1, Figs 3 and 4 and Supplementary Fig. S1). A very few monitoring schemes were implemented before or near the onset of major anthropogenic pressures, e.g. mammals in 1538, and birds and plants in 1634 (Table 1, Figs 2a and 4) but these mostly entailed non-systematic monitoring approaches or covered relatively small spatial extents.

### EBV classes and type of data collected

Comparisons of starting years among EBV classes and types of data collected were only possible for a reduced set of monitoring schemes (see Methods). Although using this restricted set meant ignoring some of the oldest schemes, the overall picture of the start of monitoring schemes dating back to the mid 1990’s is consistent with the findings resulting from all databases previously found for the taxonomic groups (Table 1 and Fig. 2).

The monitoring schemes have targeted 4 out of the 6 EBV classes from the EBV framework^24^: Genetic Composition, Species Populations, Species Traits and Community Composition. The types of data collected in the monitoring schemes include abundance of individuals (count), records of species’ presence/absence (occurrence), capture-mark-recapture data (CMR), phenological events (phenology) and measures of the population structure (population structure).

**Figure 5:**
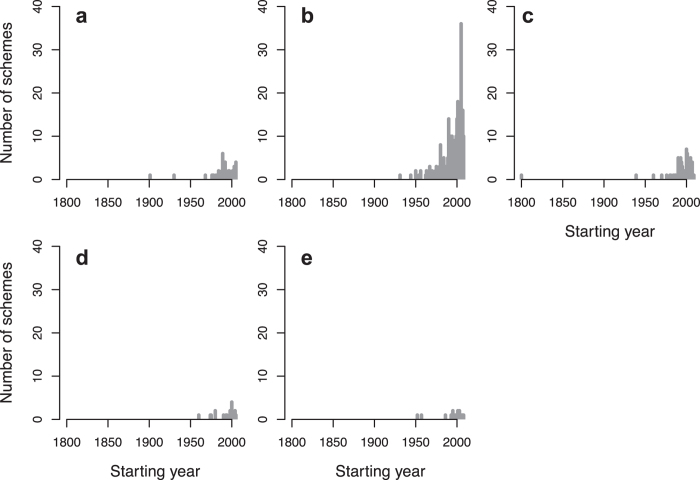
Number of monitoring schemes initiated over time according to their starting year for each type of data collected: (**a**) CMR (N = 43), (**b**) Count (N = 300), (**c**) Occurrence (N = 72), (**d**) Phenology (N = 21) and (**e**) Population structure (N = 16).

Starting years of biodiversity monitoring schemes differed among the types of data collected (Chi^2^_4_ = 10.422, p = 0.034, N = 452; Fig. 2c). Even though the oldest schemes collected CMR data (Table 1, Fig. 2c and Supplementary Table S2), the focus shifted towards the collection of count data from the 1950’s onwards (Fig. 5). Overall, the majority of the information available from biodiversity monitoring schemes are count data (66.4%, N = 300) and, to a lesser extent, occurrence data (15.9%, N = 72; Table 1; see also Supplementary Fig. S2). In comparison, data on phenology and population structure are collected in only 4.6% (N = 21) and 3.5% (N = 16) of the schemes, respectively (Table 1, Fig. 5 and Supplementary Fig. S2).

**Figure 6:**
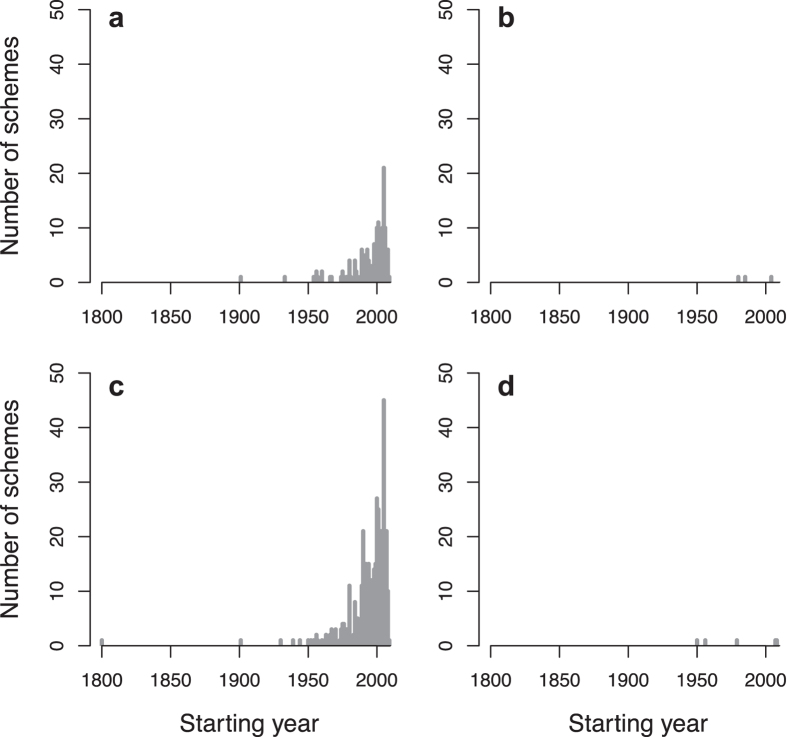
Number of monitoring schemes initiated over time according to their starting year for each EBV class targeted: (**a**) Community composition (N = 164), (**b**) Genetic composition (N = 3), (**c**) Species population (N = 433) and (**d**) Species traits (N = 5).

We did not find any significant difference in the starting years of the monitoring schemes among the EBV classes targeted (Chi^2^_3_ = 2.271, p = 0.518, N = 605; Fig. 2b). However, biodiversity monitoring schemes have focussed disproportionately on only two EBV classes: Species Populations (71.6%, N = 433) and Community Composition (27.1%, N = 164; Table 1). In contrast, the EBV classes Species Traits and Genetic Composition have been the focus of only a very small number of schemes (respectively 0.8%, N = 5 and 0.5%, N = 3; Fig. 6).

## Discussion

We provide here a first quantitative evaluation of the limitations of setting temporal baselines to fully assess the impact of major anthropogenic pressures on biodiversity. Our analysis shows that structured biodiversity monitoring data in Europe do not date back far enough in time to document and assess the full impact of anthropogenic pressures on biodiversity, even for popular taxonomic groups such as birds and mammals. Major anthropogenic pressures have continuously accelerated and escalated since the Quaternary period^13^,^15^, most remarkably during the Industrial Revolution in the middle of the 19^th^ century and from the “Great acceleration” in the 1950’s^16^,^27^. Species extinction rates reported during the last decades are considered to be comparable to those of an extinction crisis^28^. Nevertheless, extinction rates in vertebrates had exceeded the background rates as early as the 18^th^ and 19^th^ centuries, and even before this for some mammal and bird groups^29^. We demonstrate that most of the data currently available from European biodiversity monitoring schemes have been collected from the 1950’s onwards, i.e. long after modern anthropogenic pressures might have started to impact species populations and communities^29^,^30^,^31^. The sharp increase in the number of monitoring schemes from the 1990’s likely reflecting a response to the reporting commitments outlined in the European Nature Directives^32^,^33^ or similar obligations from international conventions, such as the Convention on Biological Diversity or the Convention for Migratory Species^34^. Our findings are line with previous studies showing that structured biodiversity monitoring schemes have been recently implemented^11^,^12^,^35^ and that accurate biodiversity data for major realms is not available before the 1960’s (marine^12^,^14^,^20^,^36^, terrestrial or freshwater^9^,^10^,^37^). Despite biodiversity monitoring schemes contributing to an increased understanding of recent anthropogenic impacts, the changing states of biodiversity since the rise of these pressures are mostly unknown and might be seriously underestimated^28^,^38^.

Beyond the time-series limitations of biodiversity monitoring, our analysis further illustrates a range of different sources of heterogeneity that can further diminish the relevance of available biodiversity data. We implicitly assume in this study that the starting year of monitoring schemes can be considered as a surrogate of the past states of biodiversity to document changes over time. This statement supposes a temporal continuity in monitoring, implying that any scheme ever started is still running today and that there is no temporal gap in the time-series. In practice, however, available biodiversity datasets are, at best, fragmented^37^ and most schemes are conducted on a relatively short-term basis^10^,^39^ (mean duration of schemes in this study = 15.42 ± 16.34 years, N = 452). Similarly, most biodiversity monitoring schemes are conducted at small geographical scales^9^,^10^ so that opportunities to assess past states of biodiversity at global, regional or even national scales remain limited. In addition to limited temporal coverage, inconsistencies in the temporal and spatial continuity of biodiversity monitoring schemes may therefore impose critical constraints for the assessment of biodiversity change over time.

Our analysis also highlights different sources of heterogeneity among biodiversity monitoring schemes, such as the biased representation of some taxonomic groups, the collection of only a few types of data and the relative neglect of several EBV classes. Therefore, in addition to being limited in time, the available data only reflect a fraction of the biodiversity. Existing biases in taxonomic coverage are known limitations that prevent the assessment of the changing state of the whole of biodiversity^19^,^20^,^40^, but the biases within the types of data collected or biological organisation levels that are the focus of monitoring schemes are much less frequently reported. Even if the emphasis on count and occurrence data does not systematically translate into a bias among EBV classes, the data collected in biodiversity monitoring schemes disproportionately document only two EBV classes (‘Species Populations’ and ‘Community Composition’), and overlook other EBV classes, such as ‘Species Traits’ and ‘Genetic Composition’.

Altogether, irregular temporal coverage and biases in taxonomic groups, types of data collected and EBV classes targeted offer a very truncated picture of biodiversity. Limited temporal coverage only allows a limited subset of the changing state of biodiversity needed to represent the full impact of anthropogenic pressures to be documented^41^. Besides, the majority of available biodiversity information remains inconsistent and incomplete for accurate and consistent estimates of past^12^,^17^ and changing states of biodiversity across taxa or biological organisation levels. This may promote asymmetries in biodiversity assessments and conservation objectives. For instance, if a temporal baseline was to be drawn from available data, the baseline for birds, mammals and fish would have to be set further in the past compared to reptiles, amphibians or molluscs. Consequently, previous global biodiversity assessments have been forced to use various temporal baselines^41^. In addition, the lack of consistent information about past biodiversity states is likely to maintain vagueness and promote the shifting baseline reference syndrome^4^,^5^,^6^ by creating uncertainty about past states of biodiversity^14^,^42^. Altogether, the temporal limitations and bias in biodiversity monitoring data represent a risk to misinform on the actual states and trends of biodiversity in response to anthropogenic pressures and to misguide the definition of sustainable conservation objectives.

We argue that information derived solely from current biodiversity monitoring schemes is not well suited to setting relevant temporal baselines. To face this important challenge, we encourage both scientists and policy-makers to adopt a more conservative attitude toward temporal baselines for biodiversity by explicitly recognizing the uncertainties associated with current limitations. This implies acknowledging limits to our ability to document past biodiversity states from monitoring schemes, and that the changes measured from these schemes may seriously underestimate the full impact that major anthropogenic pressures have had on biodiversity. In addition, cross-disciplinary research areas such as bio-archaeology and paleo-ecology offer promising approaches to reconstructing past states and histories of biodiversity using alternative sources of information^17^,^43^,^44^. More reliable indicators of biodiversity change could be provided by integrating historical or archeological data with recent biodiversity monitoring data. Additional mobilization and digitization of biodiversity data^45^ is needed to ensure consistent available data over large spatial extents, but strengthening research efforts to improve the linkage between monitoring, archeological and historical information^17^,^43^,^44^ is also an important way forward to extend the temporal coverage of available information. These developments and a consistent integration of fragmentary information across disciplines are critical if we are to set temporal baselines for biodiversity that reflect past states of biodiversity before the rise of major anthropogenic pressures.

## Methods

### Biodiversity monitoring databases

The databases considered in this study were selected according to the following criteria: they provide meta-data on biodiversity monitoring schemes, they are representative of monitoring practices in Europe and they contain relevant information across taxa. We considered primarily the most comprehensive meta-database describing standard information on biodiversity monitoring practices in Europe (hereafter DaEuMon). DaEuMon is based on questionnaires and was compiled under the FP6-project EuMon^9^,^46^. We considered here all schemes focusing on species monitoring that were reported in DaEuMon up to 2009 (N = 452). Since DaEuMon may only report a fraction of biodiversity monitoring schemes in Europe^9^,^46^, we considered other independent sources of data documenting biodiversity monitoring schemes in order to provide the most representative overview of existing biodiversity information in Europe. We selected two additional databases with high quality control, consistent standards and compatible meta-data structure with regard to sourced references and taxonomic, temporal and spatial coverage: The Participatory Monitoring Networks in Europe database (PMN^47^) and the Global Population Dynamics Database (GPDD) Version 2.0^48^. Like DaEuMon, the PMN database has been compiled within the FP6-project EuMon. The PMN database gathered information related to biodiversity monitoring schemes in Europe (N = 326) based on a different questionnaire structure from DaEuMon, with a very marginal overlap of schemes between the two databases. The GPDD database is one of the largest, freely available databases on species population dynamics worldwide, from which we considered only schemes conducted in Europe (see Supplementary Methods; N = 177). We combined the different biodiversity monitoring schemes from the three meta-databases whenever data interoperability allowed (see below for details).

### General approach and assumptions

We considered the starting year of each biodiversity monitoring scheme as a surrogate of the oldest state of biodiversity that can be estimated from that scheme. We broke these metrics down with respect to the (i) taxonomic group studied (ii) type of data collected (e.g. species occurrence record or count) and (iii) EBV class targeted by the schemes (for a comprehensive description of the EBV considered within each of the EBV classes see ref. 49).

Including the PMN and GPDD databases helped to improve the comprehensiveness of biodiversity monitoring when compared to the use of DaEuMon only. Combining the different databases helped counterbalance potential biases in each individual database in terms of temporal, geographical and taxonomic coverage (see Supplementary Methods and Supplementary Fig. S1 for the taxonomic coverage). Nevertheless, integrating complementary information was only possible for the comparison between taxonomic groups due to limitations in data interoperability between the three databases. As the three databases partially differed in terms of taxonomic resolution – for example, plants were mostly mentioned as “Plants” within PMN, and as “Orchids”, “Mosses, liverworts & ferns” and “Other plants” in DaEuMon – we aggregated schemes to the lowest common taxonomic level of the three databases for each taxonomic group. In contrast to taxonomy, there was no information available about the EBV class targeted and the type of data collected in PMN. In addition, GPDD almost exclusively contains biodiversity monitoring schemes that have collected count data and that have targeted the EBV class ‘Species Populations’ (specifically through the EBV ‘Population abundance’). The comparison between data types and EBV classes was thus not possible from the PMN database, and integrating information from GPDD would have strongly skewed the analysis toward one type of data and one EBV class. Consequently, the comparison between the types of data collected and the EBV classes targeted by biodiversity monitoring schemes in Europe was only carried out using DaEuMon. PMN and GPDD push back the starting years of biodiversity monitoring schemes compared to the use of DaEuMon only, but the latter provides the most representative and comprehensive overview of biodiversity monitoring practices in Europe.

Nevertheless, it is possible that the number of monitoring schemes collecting data on phenology and focusing on the EBV classes ‘Species Traits’ and ‘Genetic Composition’ are under-represented in DaEuMon. However, most trait or DNA databases do not contain structured monitoring data that allow documenting changes over time and are restricted to specific taxonomic groups (e.g. Polytraits for marine polychaetes^50^ or YouTHERIA^51^ for mammals). While trait-based monitoring databases documenting changes over time do exist, they remain scattered, difficult to access and to our knowledge are not currently compiled in any meta-database, so that such trait-based monitoring databases could not be considered in this study.

### Analysis

For each taxonomic group studied, type of data collected and EBV class targeted, we calculated descriptive metrics of the temporal baseline that could be drawn for biodiversity based on the starting year of the biodiversity monitoring schemes in Europe (median, mean, minimum or maximum). We then compared the start of biodiversity monitoring schemes with global or regional long-term time-series reflecting the major anthropogenic pressures that are known to impact biodiversity the most^1^: global human population size^52^, European temperature anomalies^53^, global land use changes^54^,^55^, global anthropogenic nitrogen and phosphorus^56^, atmospheric concentration of carbon dioxide^57^ and contaminant emissions in the United Kingdom (furan and dioxin^58^, considered as representing emissions in other European countries). In order to provide a quantitative assessment of the mismatch between the start of biodiversity monitoring schemes and the onset of anthropogenic pressures but without making any assumption about the causal relationship between the pressure and its impact on biodiversity, we here report the level that each pressure had already reached when biodiversity monitoring schemes were initiated. We first identified the value of the pressure *p*_*i*_ corresponding to the starting year of each scheme *i* by projecting the intersect between the starting year of the scheme *i* and the regression trend of the pressure on the pressure axis (see Supplementary Figure S3). We then determined the level of pressure reached at that time, expressed as the percentage of the pressure range already reached when the schemes started, as follows:


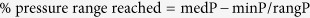


where the medP is the median of all *p*_*i*_, minP is the minimum value of the pressure over time and rangP is the known range of that pressure, which was calculated as the difference between the maximum and minimum values of the pressure along the time-series.

We used a non-parametric Kruskal-Wallis test to assess differences between the different categories considered in the biodiversity monitoring schemes (i.e. taxonomic groups studied, types of data collected and EBV classes targeted). For categories in which significant heterogeneity was found using the Kruskal-Wallis test, we performed a post-hoc analysis using the Conover-Iman multiple pair-wise comparisons test^59^. Adjustments of multiple pair-wise comparisons were made using the Benjamini-Hochberg procedure controlling for false discovery rate, which are more reliable than classical Bonferoni procedures^60^. All statistical analysis were performed using the R software^61^ (including the package *conover.test* for post-hoc analysis). Importantly, a single biodiversity monitoring scheme may have included several taxonomic groups, collected different types of data or targeted several EBV classes, and information might have been provided for some components of the questionnaires but not for others within a single monitoring scheme. Therefore, the number of monitoring schemes considered may differ between the different topical comparisons as well as the total number of schemes contained in the three databases.

## Additional Information

**How to cite this article**: Mihoub, J.-B. *et al*. Setting temporal baselines for biodiversity: the limits of available monitoring data for capturing the full impact of anthropogenic pressures. *Sci. Rep.* **7**, 41591; doi: 10.1038/srep41591 (2017).

**Publisher's note:** Springer Nature remains neutral with regard to jurisdictional claims in published maps and institutional affiliations.

## Supplementary Material

Supplementary Information

## Figures and Tables

**Table 1:** Temporal baselines of biodiversity monitoring schemes in Europe: the summary statistics of the starting years for the schemes are described for each of (a) the taxonomic groups studied, (b) the EBV classes targeted and (c) the type of data collected.

	Min.	1st Qu.	Median	Mean	3rd Qu.	Max.	N
*Taxonomic group*							
Amphibians	1817	1974	1994	1983	2000	2008	155
Birds	1634	1962	1986	1974	1998	2007	458
Fish	1634	1962	1979	1971	1994	2008	154
Insect	1804	1966	1989	1977	2000	2009	265
Mammals	1538	1964	1990	1974	2000	2008	339
Mollusc	1907	1971	2001	1981	2002	2008	18
Plants	1634	1974	1993	1975	2000	2008	245
Reptiles	1817	1982	1993	1982	2000	2008	51
**Overall**	**1538**	**1964**	**1990**	**1976**	**1999**	**2009**	**1685**
*EBV class*							
Genetic Composition	1980	1982	1985	1990	1994	2004	3
Community Composition	1901	1991	2000	1995	2005	2009	164
Species Populations	1800	1990	1999	1994	2004	2009	433
Species Traits	1950	1956	1979	1980	2007	2008	5
**Overall**	**1800**	**1990**	**1999**	**1995**	**2004**	**2009**	**605**
*Type of data*							
Capture Mark Recapture	1901	1988	1992	1989	2000	2005	43
Count	1933	1990	1999	1995	2005	2008	300
Occurrence	1800	1993	2000	1994	2003	2009	72
Phenology	1960	1990	1999	1993	2000	2005	21
Population Structure	1952	1994	2000	1994	2004	2008	16
**Overall**	**1800**	**1990**	**1999**	**1994**	**2004**	**2009**	**452**
