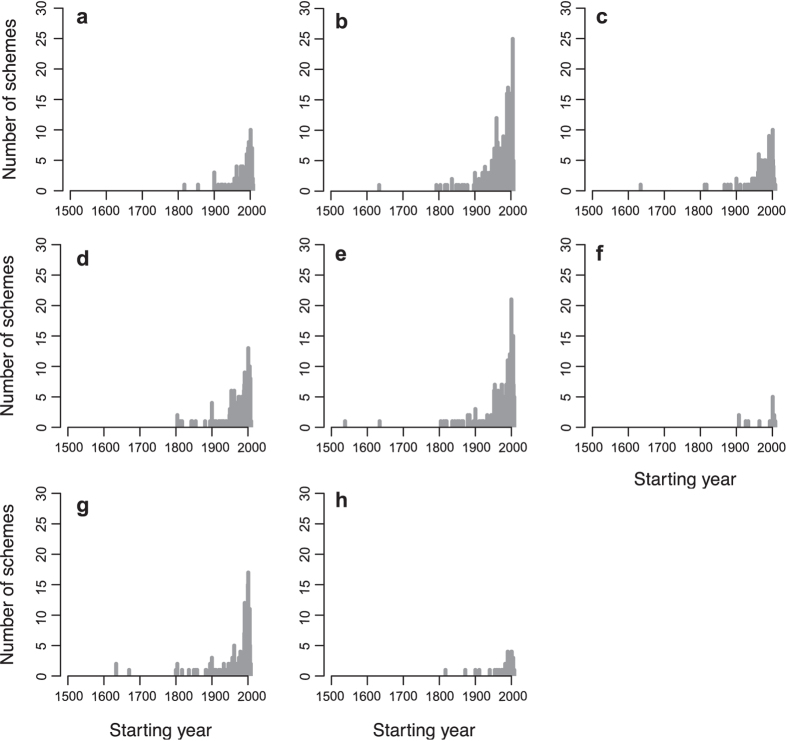# Erratum: Setting temporal baselines for biodiversity: the limits of available monitoring data for capturing the full impact of anthropogenic pressures

**DOI:** 10.1038/srep46781

**Published:** 2017-05-02

**Authors:** Jean-Baptiste Mihoub, Klaus Henle, Nicolas Titeux, Lluís Brotons, Neil A. Brummitt, Dirk S. Schmeller

Scientific Reports 7: Article number: 4159110.1038/srep41591; published online: 01 30 2017; updated: 05 02 2017

In this Article, Figure 4 is a duplication of Figure 5. The correct Figure 4 appears below as Figure 1. The legend of Figure 4 was correct from the time of publication.

## Figures and Tables

**Figure 1:**