# Supplementary material for: Setting temporal baselines for biodiversity: the limits of available monitoring data for capturing the full impact of anthropogenic pressures
Source: Sci Rep. 2017 Jan 30;7:41591. doi: 10.1038/srep41591 (PMC5278508; doi:10.1038/srep41591)
Supplement: Supplementary Information [file srep41591-s1.pdf]

Supplementary information for

Setting temporal baselines for biodiversity: the limits of available monitoring data for capturing the full impact of anthropogenic pressures

Jean-Baptiste Mihoub[1,2*], Klaus Henle[1], Nicolas Titeux[3,4] , Lluís Brotons[3,4,5], Neil A. Brummitt[6] & Dirk S. Schmeller[1,7,8]

1. UFZ-Helmholtz Centre for Environmental Research, Department of Conservation Biology, Permoserstr. 15, 04318 Leipzig, Germany
2. Université Pierre et Marie Curie, CESCO, UMR 7204 MNHN-CNRS-UPMC, Paris, France
3. European Bird Census Council (EBCC) and Forest Sciences Centre of Catalonia (CEMFOR-CTFC), InForest Joint Research Unit (CSIC-CTFC-CREAF), Ctra. Sant Llorenç de Morunys km 2, 25280 Solsona, Spain
4. Centre de Recerca Ecològica i Aplicacions Forestals (CREAF), 08193 Cerdanyola del Vallés, Spain
5. Consejo Superior de Investigaciones Científicas (CSIC), 08193 Cerdanyola del Vallés, Spain
6. Department of Life Sciences, The Natural History Museum, Cromwell Road, South Kensington, London SW7 5BD, UK
7. CNRS, EcoLab, 31062 Toulouse, France
8. Université de Toulouse, UPS, INPT, EcoLab (Laboratoire Ecologie Fonctionnelle et Environnement), 118 route de Narbonne, 31062 Toulouse, France

*Corresponding author: UMR 7204 MNHN-CNRS-UPMC, 61 rue Buffon, 75005, Paris, France. Tel. +33 1 40 79 57 61. Email: jean-baptiste.mihoub@mnhn.fr

Supplementary Methods

Selection of biodiversity monitoring schemes in the Global Population Dynamic Database

In addition to DaEuMon and the Participatory Monitoring Networks in Europe database (PMN), the Global Population Dynamic Database (GPDD) was used in this study to measure the temporal baseline for biodiversity monitoring in Europe. The GPDD compiles population time-series data such as counts, indices, or percentage cover at the global scale. To enable comparison with the DaEuMon and PMN databases, we selected from GPDD only those monitoring schemes conducted in Europe ("Europe" as "Continent" entry, with the exclusion of Russian Federation and Belarus as "Country") and which have recorded population counts and harvest catches as data type ("SamplingProtocol" filtered as "Count" and "Harvest ").

Bias associated with biodiversity monitoring databases

Geographical and taxonomic bias has been identified in DaEuMon [1] and in GPDD [2]. For instance, DaEuMon under-represents fish, while GPDD partly corrects for this taxonomic bias (DaEuMon: 3%, GPDD: 19%, PMN: 10%, total in the three databases: 9%). In contrast, GPDD under-represents amphibians and plants, while these biases are reduced when GPDD is combined with PMN and DaEuMon (for Amphibians: GPDD: 1%, DaEuMon: 6%, PMN: 13%, total in the three databases: 9%; for Plants: GPDD: less than 1%, DaEuMon: 15%, PMN: 18%, total in the three databases: 15%, see Suppl. Fig. 1). Despite such heterogeneity, general conclusions on the starting years of monitoring schemes and their relative taxonomic representation remained consistent overall between all data sources (i.e. a strong under-representation and very recent starts for reptiles and molluscs, and to a lesser extent for plants and insects).

Supplementary Table S1. Level that anthropogenic pressures had already reached when biodiversity monitoring started for each taxonomic group. The level of each anthropogenic pressure is expressed as a percentage of the known range of that pressure (see Methods for details).

**Anthropogenic pressures**	**Taxonomic groups**							
	Amphibian	Birds	Fish	Insect	Mammals	Molluscs	Plants	Reptiles
CO2	72.71	63.01	56.30	66.88	68.01	81.53	71.52	71.52
Temperature	77.69	68.62	62.06	72.29	73.35	85.59	76.60	76.60
Human	68.14	57.59	50.69	61.70	62.94	78.38	66.79	66.79
Forest	88.82	84.23	80.28	85.94	86.51	92.89	88.82	88.24
Pasture	82.43	75.63	70.81	78.36	79.16	88.50	81.60	81.60
Crop	82.02	75.09	70.19	77.87	78.68	88.22	81.17	81.17
Furan	100.00	100.00	100.00	100.00	100.00	100.00	100.00	100.00
Diox	70.73	58.37	50.48	63.15	64.59	83.03	69.14	69.14
Nitrogen	66.89	46.42	35.37	53.87	56.23	91.32	64.03	64.03
Phosphorus	74.91	64.24	57.07	68.44	69.69	85.00	73.57	73.57

Supplementary Table S2. Post-hoc multiple pair-wise comparisons for categories with significant heterogeneity. The t-test statistics and the p-values of single pair-wise comparisons performed with the Conover-Iman test adjusted through the Benjamini-Hochberg procedure are reported for categories showing heterogeneity after a significant Kruskal-Wallis test (see Methods). Significant pair-wise differences are indicated in bold.

		**Taxonomic groups**						
		Amphibian	Birds	Fish	Insect	Mammals	Molluscs	Plants
Birds	*t-test*	-3.35						
	p-val	**0.003**						
Fish	*t-test*	-3.87	-1.38					
	p-val	**0.002**	0.112					
Insect	*t-test*	-1.73	1.77	2.61				
	p-val	0.078	0.077	**0.018**				
Mammals	*t-test*	-1.94	1.73	2.59	-0.15			
	p-val	0.062	0.074	**0.017**	0.439			
Molluscs	*t-test*	1.15	2.48	2.91	1.89	1.96		
	p-val	0.154	**0.020**	**0.009**	0.064	0.065		
Plants	*t-test*	-0.45	3.36	3.83	1.46	1.69	-1.36	
	p-val	0.353	**0.004**	**0.001**	0.107	0.075	0.112	
Reptiles	*t-test*	0.24	2.37	2.96	1.40	1.50	-0.90	0.55
	p-val	0.422	**0.025**	**0.009**	0.114	0.103	0.215	0.328

		**Type of data**			
		CMR	Count	Occurrence	Phenology
Count	*t-test*	-2.97			
	p-val	**0.015**			
Occurrence	*t-test*	-2.49	0.03		
	p-val	**0.032**	0.4873		
Phenology	*t-test*	-0.76	1.25	1.12	
	p-val	0.320	0.265	0.219	
Population structure	*t-test*	-1.96	-0.35	-0.34	-1.12
	p-val	0.083	0.453	0.407	0.262

Supplementary Figure S1. Taxonomic bias associated with different sources of biodiversity information, shown as the relative contributions of different taxonomic groups in each source of biodiversity information used, extracted from three databases for Europe a) DaEuMon (N = 452 schemes), b) PMN (N = 326 schemes), and c) GPDD (N = 177 schemes) and d) one for North America [3]; N = 311 schemes); shown here for comparison only), or combinations of databases: e) DaEuMon, PMN and GPDD, f) all four databases.

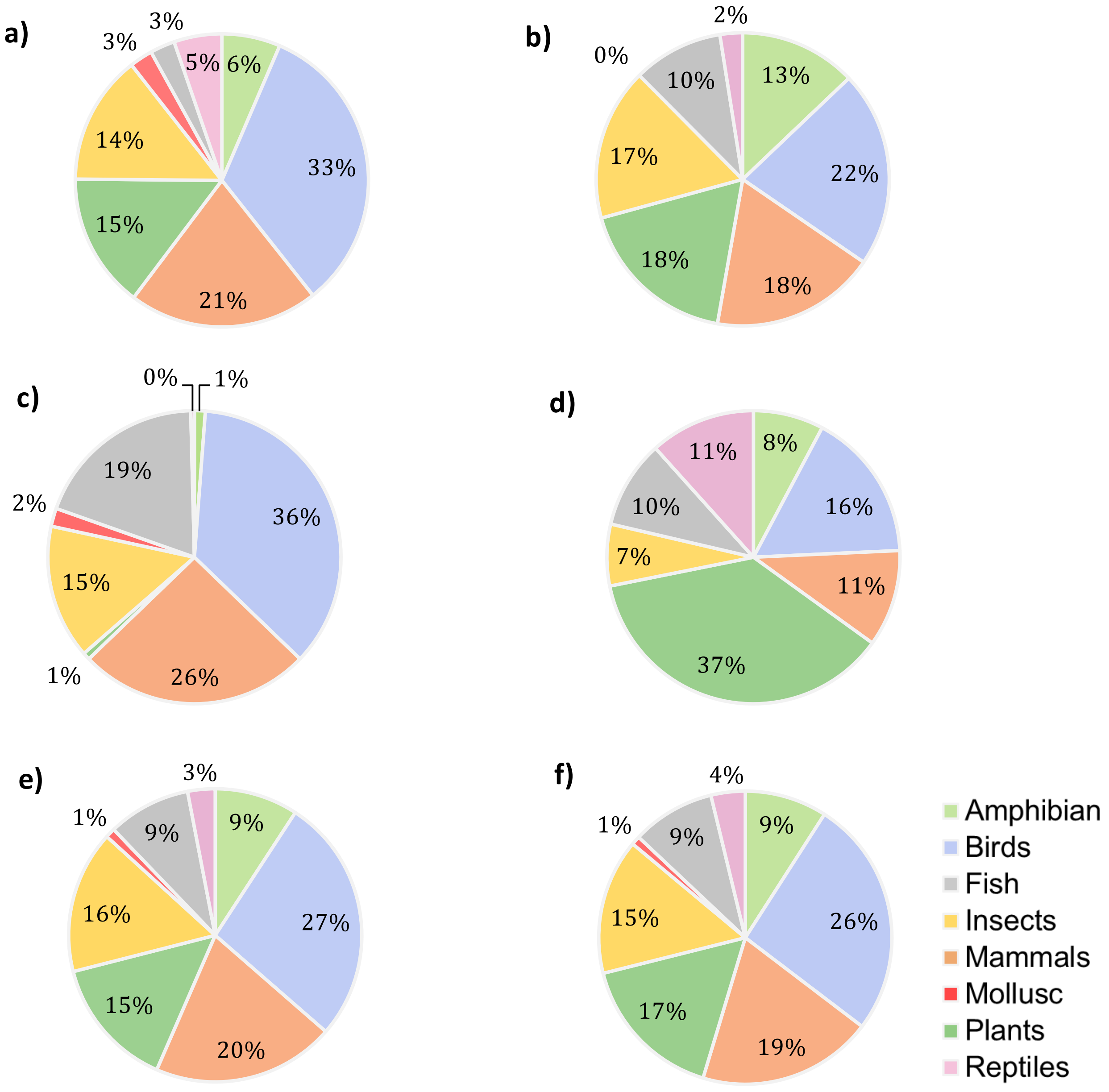

Supplementary Figure S2. Relative proportions of each data type collected by biodiversity monitoring schemes over time: per decade from 1950 onward (left) and per year from 1990 onward (right). The number of schemes considered is indicated above each bar.

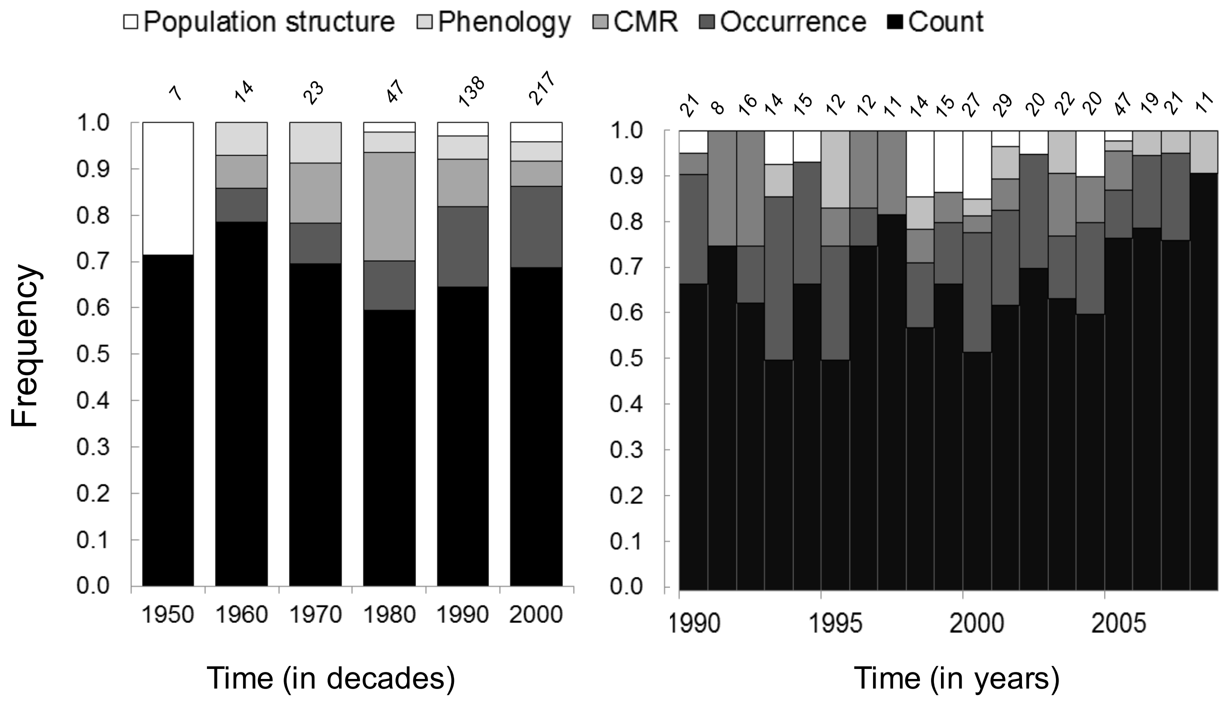

Supplementary Figure S3. Examples of the identification of the value of anthropogenic pressure when biodiversity monitoring schemes start, provided for each pressure. The intersect between the starting year of a biodiversity monitoring scheme (dashed black line) with the regression trend of the anthropogenic pressure (plain bold red line) was projected to the intersect on the pressure axis (i.e. y-axis) to determine the pressure value for that year.

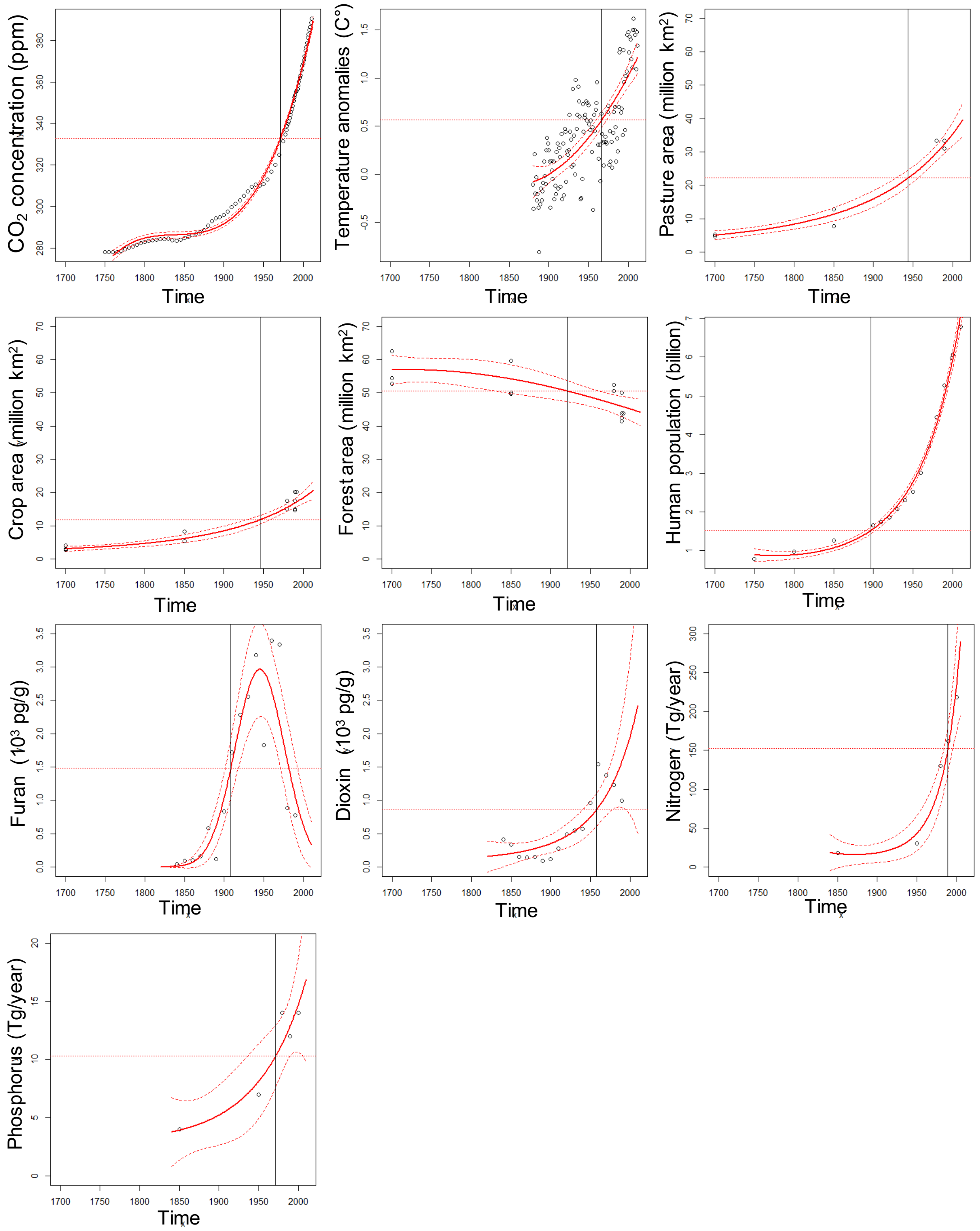

Supplementary References:

1. Schmeller D.S., Henle K, Loyau A, Besnard A, Henry P-Y. Bird-monitoring in Europe – a first overview of practices, motivations and aims. *Nat Conserv* **2**, 41-57 (2012).

2. Inchausti P, Halley J. On the relation between temporal variability and persistence time in animal populations. *J Anim Ecol* **72**, 899-908 (2003).

3. Marsh DM, Trenham PC. Current Trends in Plant and Animal Population Monitoring. *Conserv Biol* **22**, 647-655 (2008).
